# Pericapsular Nerve Group Block: An Excellent Option for Analgesia for Positional Pain in Hip Fractures

**DOI:** 10.1155/2020/1830136

**Published:** 2020-03-12

**Authors:** Utsav Acharya, Ritesh Lamsal

**Affiliations:** Department of Anaesthesiology, Tribhuvan University Teaching Hospital, Institute of Medicine, Tribhuvan University, Kathmandu, 44600, Nepal

## Abstract

Fractures in and around the hip are common presentations in the emergency department. It is commonly seen in the elderly as a result of osteoporotic changes. However, younger age groups are also affected, especially as a result of high velocity trauma. Irrespective of age, hip fractures are extremely painful, and it is difficult to position the patients for anesthesia procedures. Most of these cases are performed under subarachnoid block (SAB) or combined spinal-epidural anesthesia (CSEA), which requires the patient to be in sitting or lateral position. Here, we report a series of ten cases where pericapsular nerve group (PENG) block was administered prior to positioning the patients for SAB or CSEA. This block is a recently described regional anesthesia technique that provides excellent analgesia for hip fractures. It also provides very good analgesia for patient positioning during procedures such as SAB or CSEA.

## 1. Introduction

The femoral nerve block and fascia iliaca compartment block (FICB) are commonly used for analgesia in hip fractures. The articular branches to the hip joint originate at a higher level along the course of the nerves, so these blocks may not provide sufficient analgesia in hip fractures. The pericapsular nerve group (PENG) block is a technique that involves deposition of local anesthetic in the musculofascial plane between the psoas muscle and the superior pubic ramus [1]. This block has been recently described as an effective option for hip analgesia, as it targets the articular branches that supply the hip. It could be very useful for analgesia during the perioperative period. We report a series of ten patients who received PENG block before surgical correction of hip fractures.

## 2. Case Series

**Figure 1:**
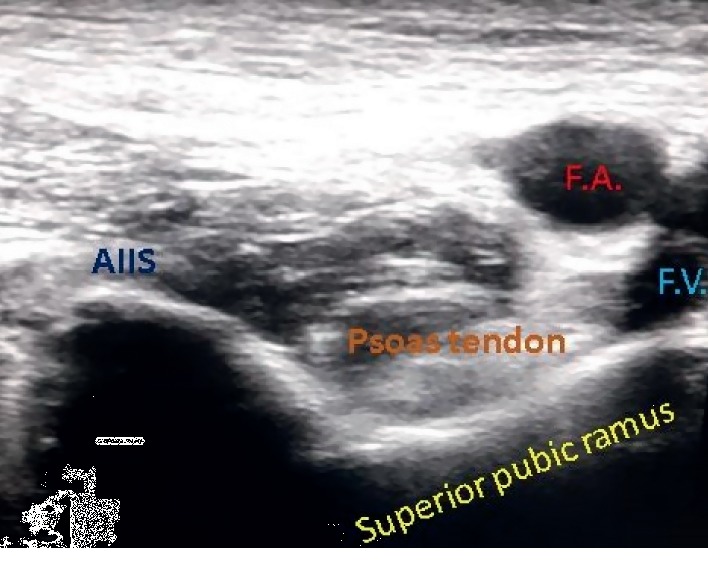
Relevant sonoanatomy for PENG block (F.A. = femoral artery; F.V. = femoral vein; AIIS = antero inferior iliac spine).

**Figure 2:**
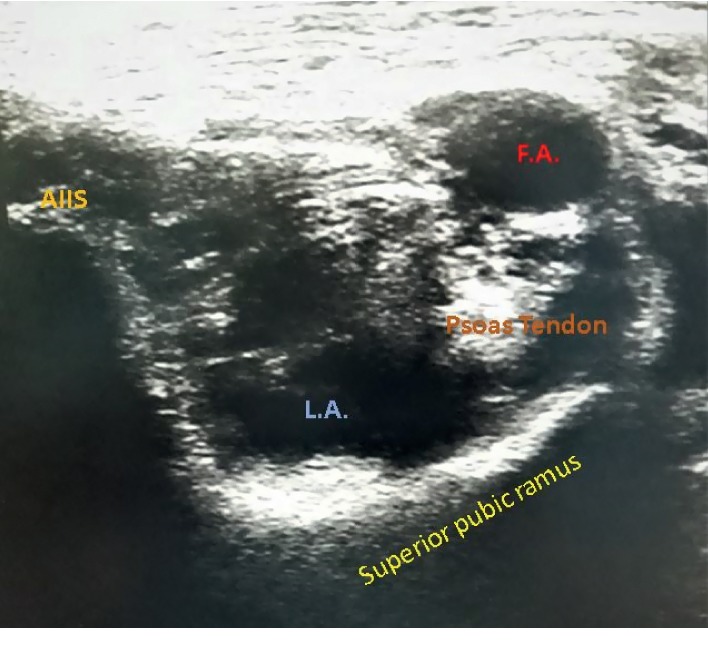
Local anesthetic spread after PENG block (L.A. = local anesthetic; F.A. = femoral artery; AIIS = antero inferior iliac spine).

The patients with hip fractures were assessed preoperatively and explained about PENG block. Written informed consent was obtained from all the patients. Pain scores during active sideways movement of the fractured limb were noted before block administration. The block was administered under ultrasound guidance with low frequency curvilinear probe. The probe was placed parallel to the inguinal crease, at the level of anterior superior iliac spine. The scanning was done with gradual caudad movement of the probe. After the anterior inferior iliac spine (AIIS) was visible, the probe was turned slightly medial until the hyperechoic continuous shadow of superior pubic ramus was visible (Figure 1). The psoas muscle with prominent tendon was then identified just above the pubic ramus. The target was the plane between these two structures. Aligning the pubic ramus in the center of the image and targeting the pubic ramus just medial to the AIIS, a standard 25G Quincke needle was introduced and 20 mL 0.125% bupivacaine with 4 mg dexamethasone was administered using ultrasound-guided out-of-plane technique. The spread of local anesthetic below the psoas tendon was noted (Figure 2). Pain scores were assessed ten minutes after the procedure and at the time of positioning for SAB.

**Figure 3:**
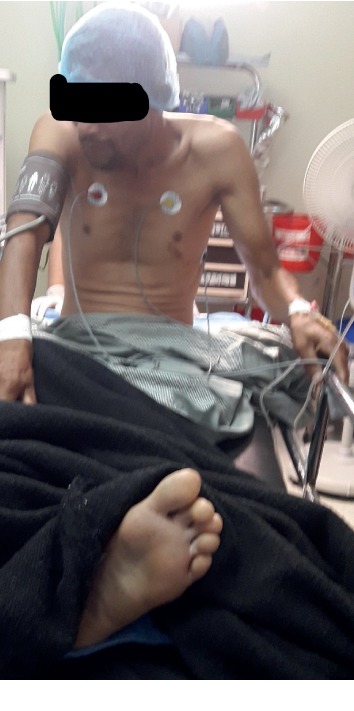
Patient with left intertrochanteric femur fracture sitting upright for spinal anesthesia after administration of PENG block.

**Figure 4:**
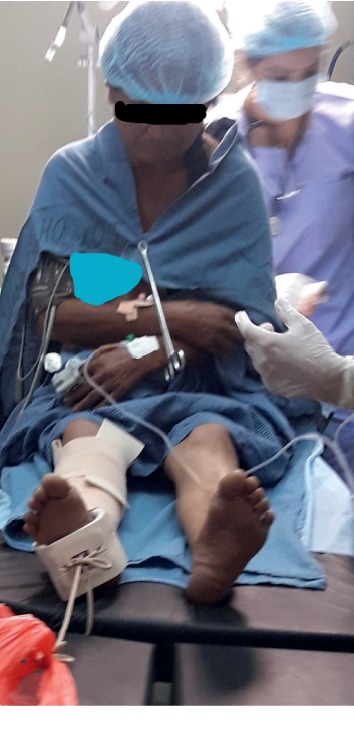
An 85-year-old lady with right intertrochanteric femur fracture maintaining upright sitting position without support after administration of PENG block.

All our patients had significant pain before PENG block. The maximum score on numeric rating scale (NRS) was 9, while the least was 6. After 10 minutes of block, the NRS scores reduced during active movement. Three patients had a score of 3, four patients had a score of 2, and three patients had a score of 1. All patients reported that they felt much better and had very little pain. At the time of SAB, nine out of ten patients sat upright without any discomfort and did not need any support (Figure 3). One 85-year-old lady needed support to sit up; however, she did not need any support to maintain the sitting position (Figure 4). The NRS score was 2 for three patients, 0 for one patient, and the rest had a score of 1 after sitting upright during the administration of SAB (Table 1).

## 3. Discussion

The nerve supply of the hip joint has been studied in detail. An anatomic study by Short et al. [2] demonstrated that high branches of both the femoral and obturator nerves provide innervation to the anterior hip capsule. It is understood that the anterior hip capsule receives the major sensory innervation, whereas the posterior and inferior capsules have no sensory fibers [3]. The accessory obturator nerve was found to innervate the medial capsule [2, 4]. In a cadaveric study, when dye was used for PENG block, it stained the entire anterior hip capsule areas related to the articular branches of femoral, obturator, and accessory obturator nerves [5]. Therefore, PENG block, theoretically, provides more complete hip analgesia among all the regional analgesia techniques so far described. Since PENG block covers the major articular branches supplying the hip joint, it is effective for pain at rest as well as during movement, such as with sitting upright.

The FICB is a relatively new component of the hip fracture analgesia armament gaining rapid popularity [6, 7]. However, there is disagreement about the exact neuroanatomy targeted by FICB [8]. Magnetic resonance imaging has revealed that the cephalad spread of local anesthetic following FICB does not consistently cover the obturator nerve. Although studies have shown FICB to provide very good analgesia in hip fracture, the recent anatomic studies of hip innervation suggest that those findings could be overstated. With the anatomical basis, PENG block is suited for more complete hip analgesia. However, large comparative studies are required to establish its superiority.

With the in-plane approach, we have found some technical difficulty, though not consistently. The AIIS comes in the needle path when introduced laterally. To avoid the AIIS, we have to adjust the needle by decreasing angulation, which then takes the needle away from the target, towards symphysis pubis. The femoral artery and vein lie in the needle path when approached from the medial side. Thus, we prefer out-of-plane approach. Regardless of the approach, one has to be precise while performing the PENG block as femoral artery and nerve lie in the vicinity. The ureter lies in close relation to the pubic ramus. Therefore, one must not go too deep if the needle is not visible clearly. Overall, this technique is relatively safe. So far, no case of vascular puncture or ureter injury has been reported.

Till date, there are no detailed studies on PENG block. In a brief report, after receiving PENG block, all five patients with hip fracture reported significantly reduced pain scores compared to baseline [1]. Similarly, our patients reported significant reduction in pain to sideways movement of the affected limb and had no discomfort in sitting and maintaining upright posture without any support.

## 4. Conclusion

The results of this case series are promising but with better understanding of the anatomy of hip innervation and the planes where the nerves lie, this new approach of nerve block may be extremely useful for analgesia in patients with hip fractures. Larger studies are warranted to validate the efficacy and superiority of PENG blocks over conventional techniques.

## Figures and Tables

**Table 1:** Characteristics before and after administration of PENG block.

Age (years)	Gender	Fracture	NRS score before PENG (on movement)	NRS score 10 minutes after PENG (on movement)	NRS score while positioning upright for spinal/epidural	Support required while positioning upright	Support required after positioning
19	M	IT	8	3	2	No	No
50	M	IT	9	1	1	No	No
45	F	ST	7	2	1	No	No
16	M	ST	6	1	0	No	No
85	F	IT	8	2	1	Yes	No
42	F	IT	7	2	1	No	No
62	F	NOF	6	1	1	No	No
52	M	IT	8	3	2	No	No
94	F	IT	9	2	2	No	No
27	M	ST	7	3	1	No	No

NRS: numeric rating scale; PENG: pericapsular nerve group; M: male; F: female; IT: intertrochanteric; ST: subtrochanteric; NOF: neck of femur.
